# Supplementary material for: Mesoporous Silica and Oligo (Ethylene Glycol) Methacrylates-Based Dual-Responsive Hybrid Nanogels
Source: Nanomaterials (Basel). 2022 Oct 30;12(21):3835. doi: 10.3390/nano12213835 (PMC9657937; doi:10.3390/nano12213835)
Supplement: Supplementary file 1 [file nanomaterials-12-03835-s001.zip › nanomaterials-1935595-supplementary.pdf]

Electronic Supplementary Information for

Micaela A. Macchione [1,2,3], Dariana Aristizábal Bedoya [2,3], Eva Rivero-Buceta [4], Pablo Botella [4,*] and Miriam C. Strumia [2,3,*]

[1] Centro de Investigaciones y Transferencia de Villa María (CIT Villa María), CONICET-UNVM, Arturo Jauretche 1555, Villa María, Córdoba X5900LQC, Argentina
[2] Departamento de Química Orgánica, Facultad de Ciencias Químicas, Universidad Nacional de Córdoba, Av. Haya de la Torre esq. Av. Medina Allende, Córdoba X5000HUA, Argentina
[3] CONICET, Instituto de Investigación y Desarrollo en Ingeniería de Procesos y Química Aplicada (IPQA). Av. Velez Sárfield 1611, Córdoba X5000HUA, Argentina
[4] Instituto de Tecnología Química, Universitat Politècnica de València-Consejo Superior de Investigaciones Científicas, Av. Los Naranjos s/n, 46022 Valencia, Spain
* Correspondence: pbotella@itq.upv.es (P.B.); mstrumia@unc.edu.ar (M.C.S.)

**Sections**	**Content**
ESI-1. Characterization of MSNs	Figure S1 and Table S1
ESI-2. Silanization Reaction of MSNs	Figure S2
ESI-3. FT-IR Analysis of Samples with Acidic Moieties	Figure S3
ESI-4. ^{1}H NMR	Figure S4
ESI-5. Transmission Electron Microscopy	Figure S5A and S5B
ESI-6. UV-Visible Calibration Curve	Figure S6
ESI-7. Release Mechanism	Figure S7 and Table S2
ESI-8. Biocompatibility and Cytotoxicity	Figure S8

ESI-1. Characterization of MSN

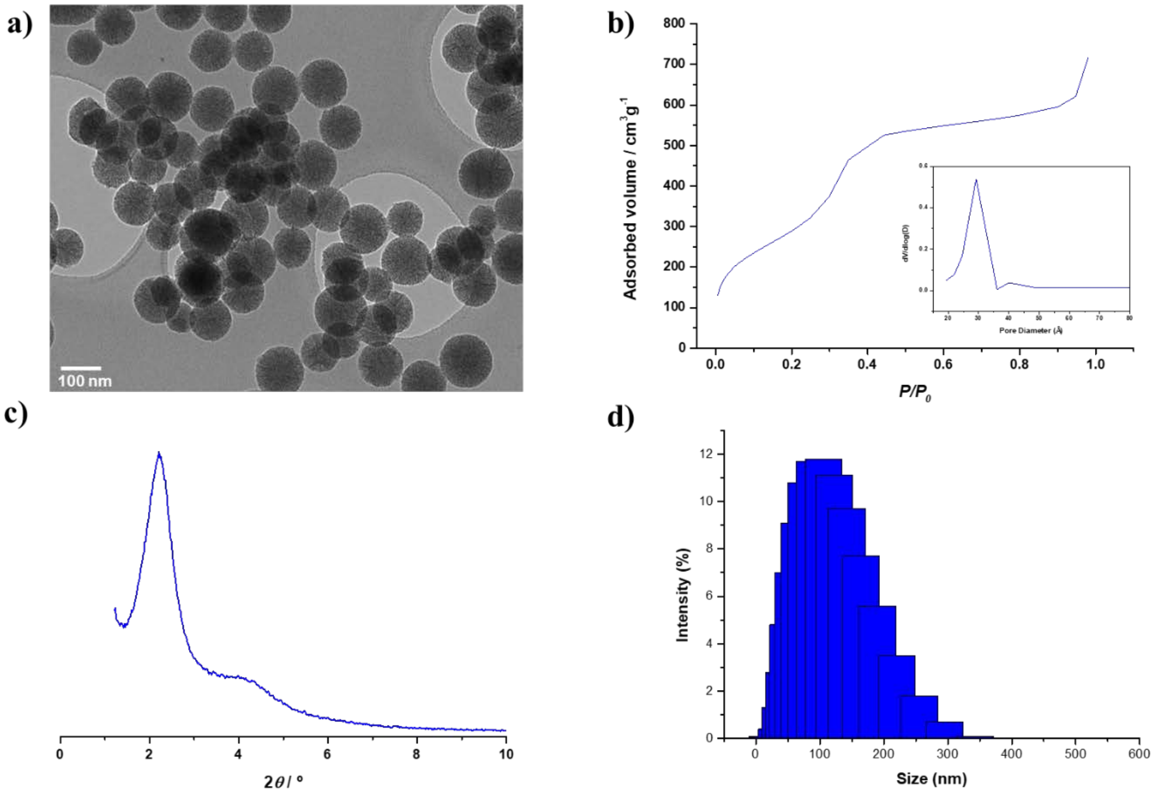

Figure S1. (a) TEM micrograph of MSNs. (b) Powder XRD pattern of MSNs. (c) Nitrogen adsorption-desorption isotherms of MSNs acquired at 77 K. The inset shows the pore size distribution of MSNs. (d) Dynamic Light Scattering (DLS) of MSNs.

Table S1. Main characteristics of MSNs tested in the present work.

	DLS[a]			N_2 physisorption		
Sample	PDI	Dh (nm)	ζ -potential (mV)	S_{BET} (m^2 g^{-1})	V_{pore} (cm^3 g^{-1})	d_{pore} (nm)
**MSNs**	0.192	92.00	-13.8	1105.98	1.02	3.96

[a]*As determined by dynamic light scattering.*

ESI-2. Silanization Reaction of MSNs

Firstly, the role of triethylamine as catalyst of the silanization reaction was evaluated. Our results show that in the absence of triethylamine, the peak of MEMO a 1720 cm^{-1} is not observed indication the functionalization is null o extremely low (Figure S2 (a)). Note that the peak observed around 1630 cm^{-1} can be attributed to MSN. Otherwise, by observing the peak of MEMO a 1720 cm^{-1}, an evidence of the functionalization is obtained in the presence of triethylamine. These results are in perfect agreement with previous reports [3].

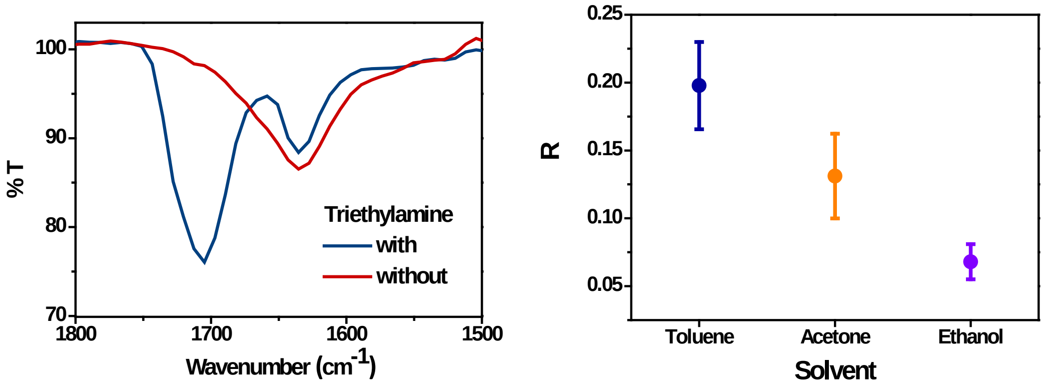

(a) (b)

Figure S2. (a) FT-IR spectra of silanization reaction with and without triethylamine. (b) Relation of maximum absorbances values from the bands: 1720 cm^{-1} of MEMO and 1100 cm^{-1} of MSNs of silanization reactions performed in toluene, acetone, and ethanol.

For the analysis of the degree of functionalization in each batch of samples, we have calculated the relation of maximum absorbances values from the band around 1720 cm^{-1} corresponding to C=O of MEMO and around 1100 cm^{-1} attributed to Si-O-Si of MSNs. Then, this relation "R" was calculated as follows:

$$R = \frac{\text{Max Abs 1720}}{\text{Max Abs 1100}}$$

The effect of the nature of the solvent was evaluated by remaining the concentration of MEMO constant (0.033 M) and employing ethanol, acetone, and toluene (Figure S2 (b)). This analysis shows that the nature of the solvent is particularly important for the silanization reaction, and higher degree of functionalization given by the relation of bands is shown in toluene as it was previously reported [4]. As a criterion to continue to the next step of the synthesis process, the relation R must be around 0.2.

ESI-3. FT-IR Analysis of Samples with Acidic Moieties

FT-IR studies were performed to determine the incorporation of these acidic moieties. The HNGs with and without acids show notable differences in the area corresponding to the carbonyl stretching (Figure S3). The maximum peak of HNG without acidic monomer is around 1733 cm-1 (aliphatic ester vibration), while HNGs with 4% of AA and IA exhibit a maximum in 1726 and 1727 cm-1, respectively. The differences observed exposed the presence of carbonyls of ester and a mixture of carbonyls of ester and carboxylic acid, respectively.

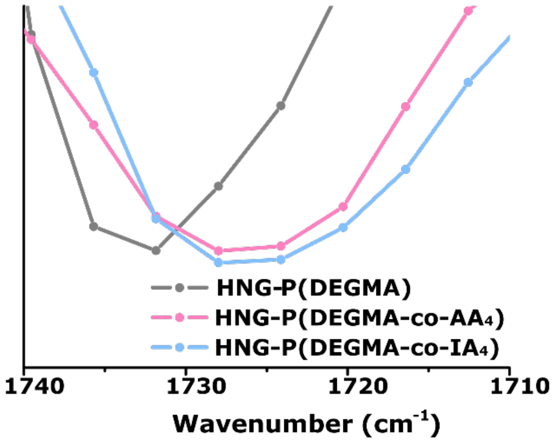

Figure S3. FT-IR carbonyl region of HNGs without acid co-monomer (grey line), with 4 % AA (pink line), and with 4 % IA (light blue line).

ESI-4. ¹H NMR

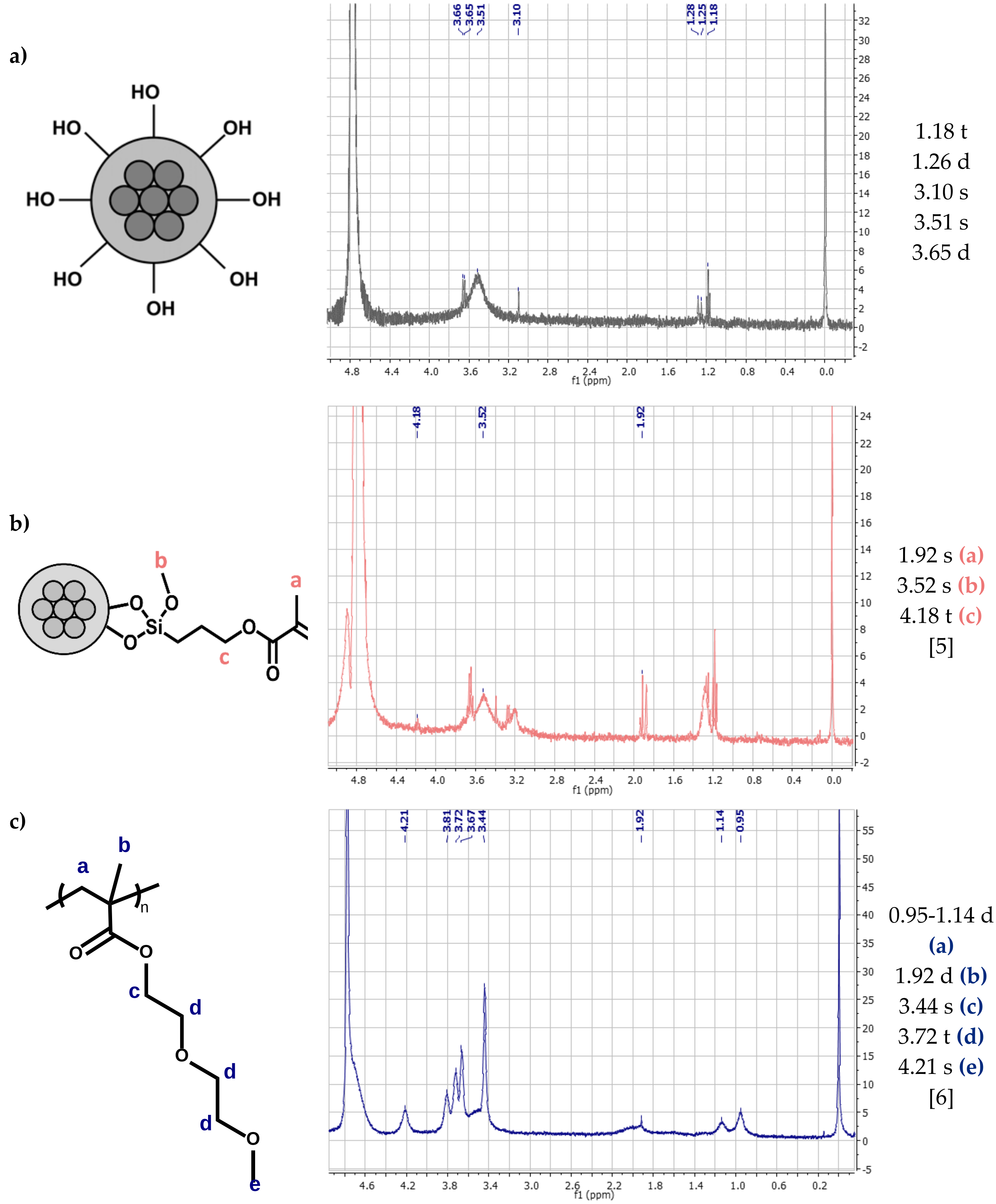

Figure S4. ¹H NMR spectra of: (a) pristine MSNs; (b) MSN@MEMO, and (c) HNG-P(DEGMA).

ESI-5. Transmission Electron Microscopy

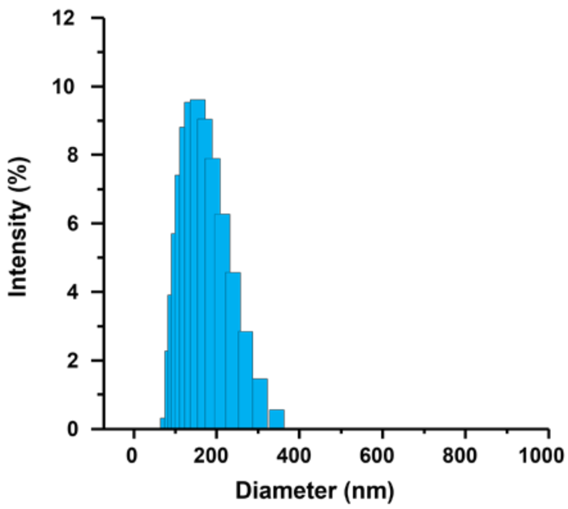

Figure S5A. Size distribution as determined from the measurement of more than 250 nanoparticles.

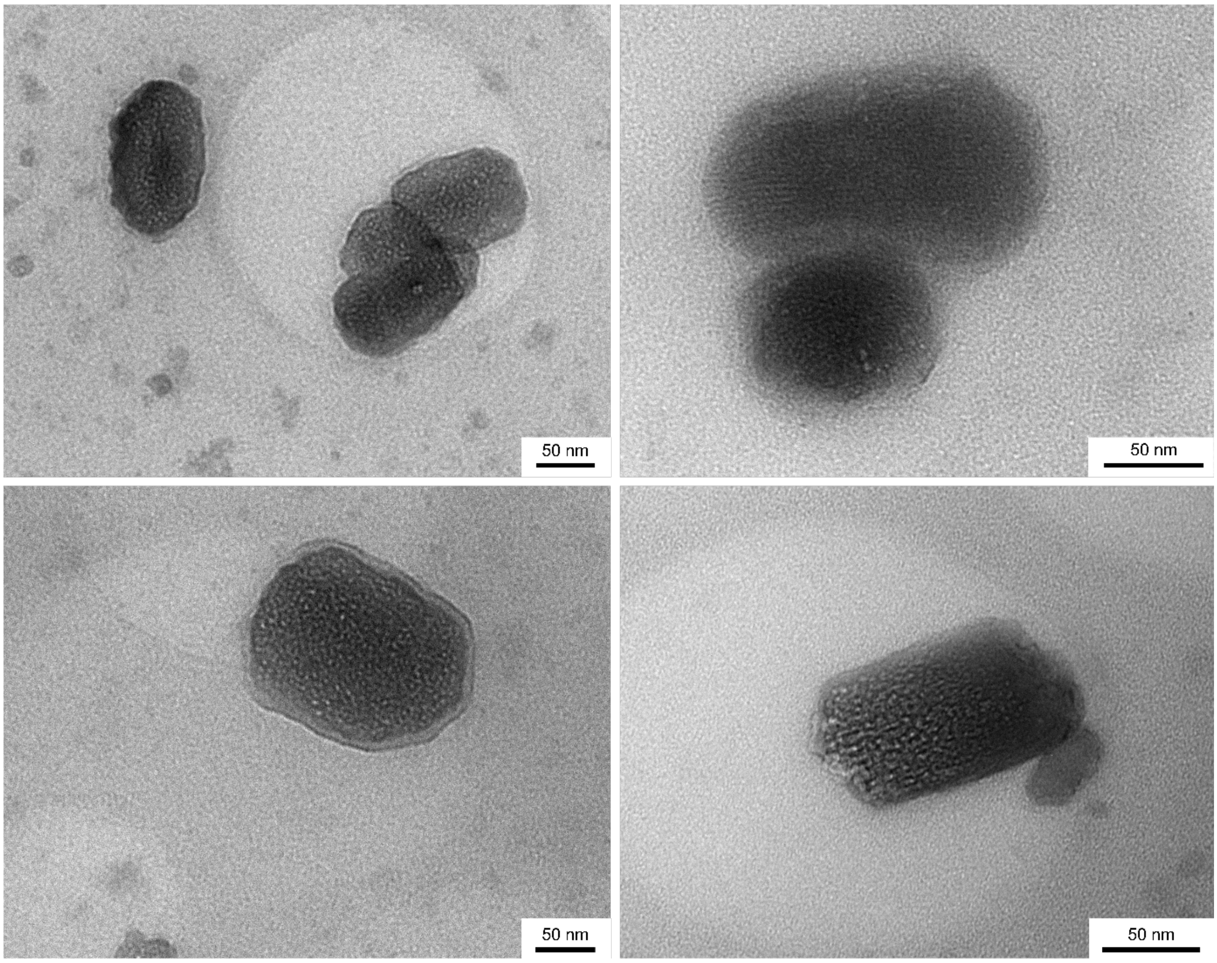

Figure S5B. High magnification TEM images of isolated HNGs (HNG-P(DEGMA-co-IA_{12})).

ESI-6. UV-Visible Calibration Curve

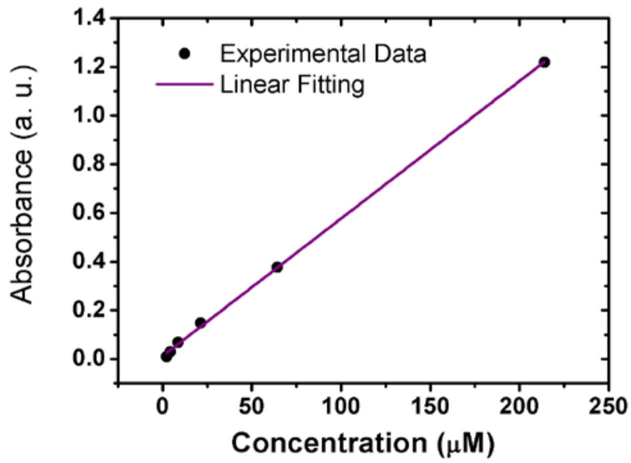

Figure S6. Calibration curve was performed in MeOH:HCl 95:5 to obtain the molar absorptivity coefficient by UV–vis spectrophotometry, measuring the absorbance at 302 nm. The following CPT concentrations were used: 2.14, 4.18, 8.6, 21.4, 64.2 and 214 μM.

ESI-7. Release Mechanism

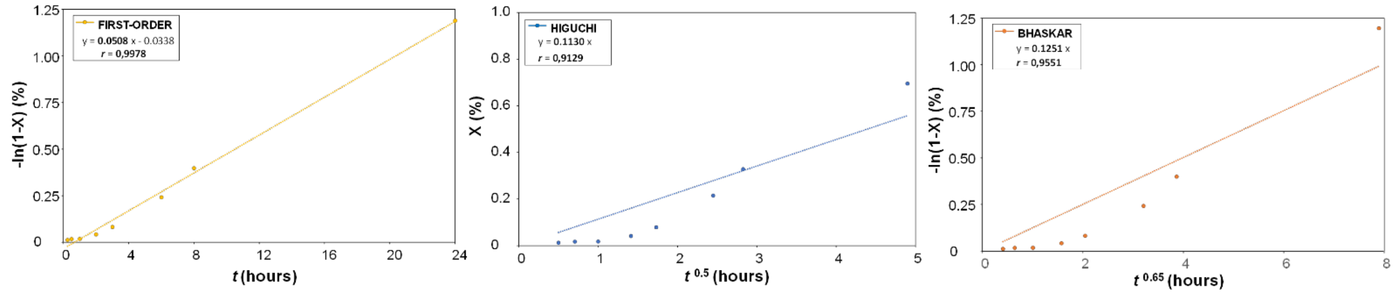

Figure S7. Plots of different kinetic models for the release of CPT from the HNG-P(DEGMA-co-IA_{12}) in PBS, at pH 7.4 and 40 ºC.

Table S2. Fitting parameters of kinetic models for CPT release from HNG-P(DEGMA-co-IA_{12}) nanoparticles in PBS, at pH 7.4 and 40 ºC.

Model	First-Order	Higuchi	Bhaskar
k[a]	0.0508	0.1296	0.1251
r[b]	0.9978	0.9129	0.9551

[a] Kinetic constant.

[b] Regression coefficient.

ESI-8. Biocompatibility and Cytotoxicity

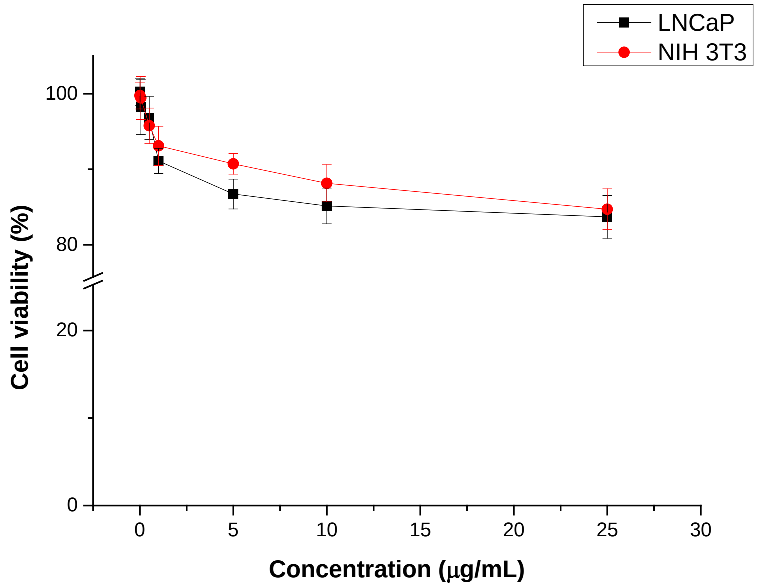

Figure S8. *In vitro* MTS cell viability assays in LNCaP and NIH 3T3 cells incubated with variable concentrations of HNG-P(DEGMA-co-IA_{12}) hybrid nanogels (mean±SEM).

References

[1] D. F. Enache *et al.*, "Schiff base-functionalized mesoporous silicas (MCM-41, HMS) as Pb(II) adsorbents," *RSC Adv.*, vol. 8, pp. 176–189, 2018.

[2] Q. Cai, Z. S. Luo, W. Q. Pang, Y. W. Fan, X. H. Chen, and F. Z. Cui, "Dilute solution routes to various controllable morphologies of MCM-41 silica with a basic medium," *Chem. Mater.*, vol. 13, pp. 258–263, 2001.

[3] K. M. R. Kallury, P. M. Macdonald, and M. Thompson, "Effect of Surface Water and Base Catalysis on the Silanization of Silica by (Aminopropyl) alkoxysilanes Studied by X-ray Photoelectron Spectroscopy and C-13 Cross-Polarization Magic-Angle-Spinning Nuclear-Magnetic-Resonance," *Langmuir*, vol. 10, no. 2, pp. 492–499, 1994.

[4] C. R. Suri and G. C. Mishra, "Activating piezoelectric crystal surface by silanization for microgravimetric immunobiosensor application," *Biosens. Bioelectron.*, vol. 11, no. 12, pp. 1199–1205, 1996.

[5] C. Bressy, V. G. Ngo, F. Ziarelli, and A. Margaillan, "New insights into the adsorption of 3-(trimethoxysilyl)propylmethacrylate on hydroxylated ZnO nanopowders," *Langmuir*, vol. 28, pp. 3290–3297, 2012.

[6] H. Dong and K. Matyjaszewski, "Thermally responsive P(M(EO)2MA- co -OEOMA) copolymers via AGET ATRP in miniemulsion," *Macromolecules*, vol. 43, no. 10, pp. 4623–4628, 2010.
